# TNF Decoy Receptors Encoded by Poxviruses

**DOI:** 10.3390/pathogens10081065

**Published:** 2021-08-22

**Authors:** Francisco Javier Alvarez-de Miranda, Isabel Alonso-Sánchez, Antonio Alcamí, Bruno Hernaez

**Affiliations:** Centro de Biología Molecular Severo Ochoa, Consejo Superior de Investigaciones Científicas, Campus de Cantoblanco, Universidad Autónoma de Madrid, Nicolás Cabrera 1, 28049 Madrid, Spain; fj.alvarez@cbm.csic.es (F.J.A.-d.M.); ialonso@cbm.csic.es (I.A.-S.); aalcami@cbm.csic.es (A.A.)

**Keywords:** poxvirus, immune evasion, tumour necrosis factor, tumour necrosis factor receptors, lymphotoxin, inflammation, cytokines, secreted decoy receptors, vaccinia virus, ectromelia virus, cowpox virus

## Abstract

Tumour necrosis factor (TNF) is an inflammatory cytokine produced in response to viral infections that promotes the recruitment and activation of leukocytes to sites of infection. This TNF-based host response is essential to limit virus spreading, thus poxviruses have evolutionarily adopted diverse molecular mechanisms to counteract TNF antiviral action. These include the expression of poxvirus-encoded soluble receptors or proteins able to bind and neutralize TNF and other members of the TNF ligand superfamily, acting as decoy receptors. This article reviews in detail the various TNF decoy receptors identified to date in the genomes from different poxvirus species, with a special focus on their impact on poxvirus pathogenesis and their potential use as therapeutic molecules.

## 1. TNF Biology

TNF is a potent pro-inflammatory cytokine with a broad range of biological effects, ranging from the activation of inflammatory gene programs to cell differentiation or apoptosis induction while also playing an essential role in the host control of many viral infections [1,2]. It was originally identified from the serum of animals treated with bacterial lipopolysaccharide (LPS) as a molecule that caused the necrosis of tumours in vivo, hence its name. TNF is mainly secreted from activated macrophages, but also from natural killer and T-cells, neutrophils, eosinophils, mast cells and other non-immune cells, such as endothelial cells or neurons [3]. TNF is the best characterized member of the TNF ligand superfamily (TNFLSF), which includes 19 structurally related ligands that bind to type I membrane glycoproteins at the cell surface, grouped as TNF receptor (TNFR) superfamily (TNFRSF), to trigger TNFR signalling [4,5].

TNF is first produced as a 26 kDa class II transmembrane protein (tmTNF) on the surface of cells. Then, tmTNF is cleaved by the metalloprotease TNF-converting enzyme (TACE) to be released as a soluble homotrimeric cytokine (sTNF) of about 51 kDa that can execute its function far away from the site where it is synthesized [6]. 

**Figure 1:**
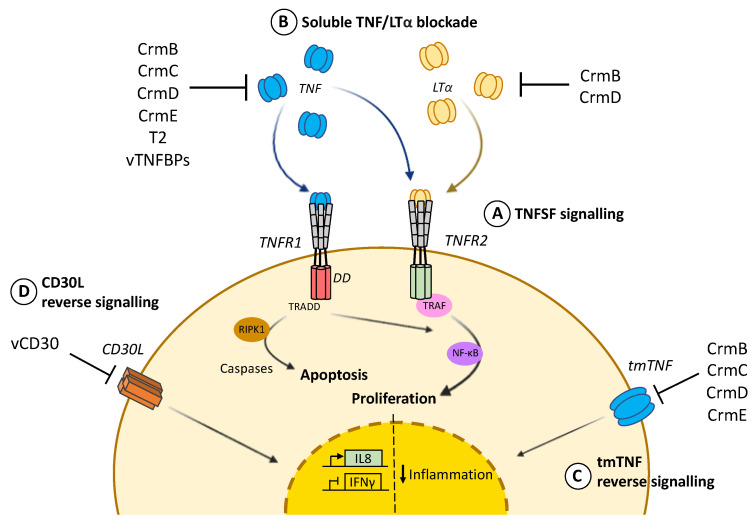
TNF cellular response and viral counteraction. (A) TNF signalling through intracellular death domains (DD) in TNFR1 recruits TRADD and triggers apoptotic cell death via RIPK1 and the caspases cascade. In contrast, prosurvival action mediated by TRAF and NF-κB can be induced through TNFR2 by either TNF or LTα. This second signalling cascade, leading to cell proliferation, can also be activated by TNFR1. (B) vTNFRs and vTNFBPs differentially bind and block TNF and LTα to prevent cytokine binding to cognate cellular receptors. (C) vTNFRs CrmB, CrmC, CrmD and CrmE can also promote reverse signalling through tmTNF at the cell surface and dampen inflammation. (D) vCD30 also induces reverse signalling through CD30L, causing inhibition of IFNγ and expression of IL8.

Far from just being a precursor, tmTNF is a key regulator for many immune and inflammatory processes, as both forms of TNF (tmTNF and sTNF) can bind to two different transmembrane cellular receptors with different affinities [7]. Whereas sTNF binds preferentially to the ubiquitous TNFR1 (also known as p55/p60), tmTNF is the main activating ligand for TNFR2 (also known as p75/p80), which is only expressed in certain endothelial and immune cell types [8]. Both cell surface TNFRs are type I membrane glycoproteins and similar in their extracellular domains involved in ligand binding, which comprise four cysteine-rich domains (CRDs), each of them containing about 40 residues including six conserved cysteines involved in the formation of internal disulfide bridges. The transmembrane domain of TNFRs is conserved through evolution and required for efficient signalling [9]. Despite these similarities, TNFR1 and TNFR2 belong to different subgroups of the TNFRSF due to the differences in their intracellular signalling domains. The TNFR1 cytoplasmic domain harbours a death domain that enables direct interaction with the TNFR-associated death domain (TRADD) in response to TNF binding. The TNFR1-TRADD complex then serves as a docking platform to recruit diverse signalling proteins such as the receptor-interacting serine/threonine-protein kinase 1 (RIPK1), that initiate apoptotic cell death via the caspase cascade. In contrast, the intracellular part of TNFR2 does not contain a death domain and promotes cell survival after TNF binding through recruitment of TNF receptor-associated proteins (TRAF) that trigger activation of transcription factor nuclear factor-kB (NF-kB). Activation of NF-κB may also occur after TNF stimulation via TNFR1, since the TRADD-TRAF2-RIPK1 complex can additionally serve as a scaffold for NF-κB essential modulator (NEMO) binding, which is essential for the inhibition of NF-kB kinase (IkB) degradation. In summary, TNF pro-survival action required for cell proliferation or differentiation can be executed either through TNFR1 or TNFR2, while TNF-mediated cell death mainly occurs through TNFR1 signalling (Figure 1). 

Soluble versions of TNFR1 and TNFR2 may also exist as a result of alternative splicing or proteolytic cleavage [10,11]. To regulate TNF biological activity, these soluble variants inhibit TNF by competing for its binding with their membrane bound counterparts, thus acting as decoy receptors. Similarly, soluble receptors have been described for other members of the TNFLSF such as decoy receptor 1 (DcR1) which competes for TRAIL or decoy receptor 3 (DcR3) which binds FasL, LIGHT and TL1A [12,13,14]. 

In addition, tmTNF can induce reverse signalling, a process by which this membrane-bound cytokine can also act as a receptor and trigger intracellular signalling in tmTNF-bearing cells. This tmTNF-elicited reverse signalling is key for the activation and regulation of T cells, macrophages and natural killer (NK) cells [15,16].

Another TNFLSF member is lymphotoxin-α (LTα), the closest homolog to TNF with 51% sequence similarity, which mainly exists as a soluble homotrimer secreted from T and B lymphocytes. LTα differs from TNF in the amino terminal part, which resembles a typical signal peptide, making LTα secretion extremely efficient. Indeed, unlike most members of TNFLSF, LTα can only anchor to the cell surface after association with membrane-bound lymphotoxin-β (LTβ) to form a heterotrimer [17]. LTα binds with high affinity to the same receptors as TNF, TNFR1 and TNFR2, although signalling is exclusively triggered through TNFR2. The role of TNFR2 binding to LTα would be related to the regulation of the biological functions of this cytokine [18]. Besides TNFR1 and TNFR2, LTα can bind to herpesvirus entry mediator (HVEM) with low affinity [19]. Phenotypes from LTα knockout mice point to a specific role of this cytokine in the development of secondary lymphoid organs, host defence against infections and inflammation, since these animals show the absence of lymph nodes and lack NK cells resulting in increased mortality against infections [20,21,22]. When challenged against an infection, these LTα knockout mice exhibited decreased cellular response and defective viral clearance [23,24]. However, it was not clear whether this phenotype was directly associated with the absence of LTα or could be a consequence of the general malfunction of secondary lymphoid organs.

### TNF Antiviral Activity

One of the main physiological roles of TNF is the mediation of acute inflammation during virus infection. TNF is locally induced early after virus infection and enhances its own expression together with other pro-inflammatory cytokines and also the secretion of chemokines that promote the recruitment and activation of leukocytes. In animal models of infection, TNF, synergistically with interferon (IFN) γ, has been shown to interfere with the replication of diverse viruses, such as hepatitis B virus [25] or murine cytomegalovirus [26], among others.

The specific relevance of TNF and TNF signalling in the protection against poxvirus infections is also largely known and supported by experimental data from in vivo studies. For instance, mouse strains naturally resistant to a lethal poxvirus infection, such as ectromelia virus (ECTV), became more susceptible after TNFR deletion, since 60% of the knockout animals succumbed to infection [27]. Indeed, treatment with mouse TNF has been shown to reduce ECTV replication and mortality to some extent [28]. In line with this, another study showed that infection of TNFR2 knockout C57BL6 mice with vaccinia virus (VACV) results in reduced liver inflammation and defective viral clearance, leading to higher viral loads compared to wild type animals [29]. The direct antiviral activity of TNF during mice infections was demonstrated in another study using a recombinant VACV expressing mouse TNF [30,31]. In this case, the ectopic expression of the cytokine resulted in a restriction of VACV growth during infection, most likely due to a massive increase in the number of neutrophils. However, perhaps the most fascinating evidence pointing to the relevance of TNF during poxvirus infections comes from the identification of diverse viral TNFRs (vTNFRs) encoded in the poxvirus genomes. As later discussed, the presence of diverse vTNFRs highlights that a viral anti-TNF action is required for virus dissemination during poxvirus infections.

## 2. Virally Encoded TNFRs and TNFBPs

Poxviruses are known for their broad repertoire of immunomodulatory proteins dedicated to evading the host response during infection. One of their most distinctive immune evasion mechanisms is the secretion of viral proteins from infected cells with the ability to bind key host cytokines before engagement with cellular receptors, thus acting as decoy receptors [32]. These proteins often represent soluble versions of their cellular homologs, indicating that they have evolved from their vertebrate hosts, while in other cases they lack homology to any known cellular protein. To specifically prevent the antiviral action of TNF, poxviruses encode two types of secreted proteins that bind diverse members of the TNFLSF: vTNFRs, which are functionally and structurally homologous to cell receptors, and novel viral TNF binding proteins (vTNFBP), with no known homology to host TNFRs (Table 1). 

### 2.1. Virally Encoded TNFRs 

vTNFRs are soluble proteins that mimicking the extracellular domains of TNFRSF and bind to members of TNFLSF in solution, preventing their interaction with cognate receptors at the cell surface (Figure 1). To date, four main vTNFRs have been described: cytokine response modifier B (CrmB), CrmC, CrmD and CrmE. In addition, Leporipoxvirus T2 protein and a viral version of TNFRSF CD30 (vCD30) complete the collection of poxviral vTNFRs (Table 1). 

Although the structure of just one vTNFR has been experimentally determined [41], it is well accepted that the N-terminal region of every vTNFR is functionally and structurally homologous to that of cellular TNFRs. These are composed of up to five CRDs that mediate TNF binding [50], while the key residues determining the ligand affinity have been mapped to CRD2 and CRD3 [51]. A recent study combining structural data with TNF and LTα binding specificities determined that the molecular ligand binding mechanism is fairly conserved and dominated by a groove under the 50s loop region in CRD2 from CrmD [52]. Although the binding mechanism displayed by CRD2 is common for many vTNFRs, these receptors appear very ligand specific due to variable determinants present in CRD3 that recognize specific structural features of their ligands [53]. Interestingly, CRD1 is not involved in ligand binding but can act as a preligand assembly domain (PLAD) and enables the self-association of TNFRs in a ligand independent manner. This mechanism allows receptors to exist in a trimeric form before recognizing their ligand and presumably enhances their affinity and signalling potency [54,55,56]. In contrast with their cellular counterparts, there is no consensus on the number of CRDs across vTNFRs. For instance, CrmB and CrmD exhibit four CRDs (CRD1 to CRD4), while CrmC and CrmE only present three of them (CRD1 to CRD3) [35,36,37,57]. Similarly, their sequence is not equally conserved, as CrmD and CrmB share more than 50% amino acid identity in their CRDs region, whereas the similarity of CrmE to any other vTNFR is approximately 45% and down to 35% in the case of CrmC, the most divergent vTNFR [36].

As stated before, vTNFRs represent soluble viral versions of the membrane-anchored cellular receptors. However, VACV CrmE can additionally associate to the cell surface, maintaining its TNF binding properties intact. Since CrmE lacks a transmembrane domain, this association might occur through interaction with cellular proteins, remaining attached to the cell surface before its cleavage to act as a soluble inhibitor. Alternatively, CrmE might be retained through its N-terminal region at the cell surface, allowing CRDs exposure to bind soluble TNF [58].

Diversity also extends to the number of gene copies encoding vTNFRs among different poxviruses. For example, while one copy of the CrmC gene is found in CPXV, CrmB and CrmD are encoded by two gene copies in CPXV and ECTV, respectively. The number of gene copies encoding the same vTNFR can even vary across poxvirus species to influence its expression levels. Remarkably, although CrmD is expressed at lower levels than CrmC by CPXV, two gene copies in ECTV provide higher expression levels of CrmD during infection [37].

#### 2.1.1. vTNFRs Affinities and Evolution

One of the most fascinating aspects of vTNFRs is their degree of specificity to bind diverse members of the TNFLSF together with their specialization to discriminate them from different animal species. CrmC [35] and CrmE [59] were first identified as specific TNF inhibitors, while CrmB [57] and CrmD [37] were shown to additionally bind and inhibit LTα effects in vitro. Surprisingly, in a comprehensive study to determine the affinities of most viral and human secreted TNFRs, LTβ was identified as a ligand for CrmB and CrmD, although it signals through a completely different receptor [5,36,60]. 

It is worth noting that vTNFRs CrmB, C, D and E are differentially distributed among poxvirus species. It seems that those poxviruses with a strict host range, like variola virus (VARV) and ECTV, usually express just one of them. For instance, a strictly human poxvirus like VARV only expresses CrmB as functional vTNFR and the mouse-specific ECTV expresses only CrmD [61,62,63]. On the contrary, some poxvirus species with a broad host range, such as CPXV, express up to four different vTNFRs [64,65]. In the case of VACV, a virus with an unclear host and undetermined origin, most strains do not express any functional TNF-binding proteins since the corresponding vTNFRs encoding genes have been eliminated or truncated [66,67]. However, the VACV strains Lister, Evans, and USSR express CrmC and CrmE [68]. This suggests that different poxvirus species have selected certain vTNFRs and this distribution could reflect their unique evolutionary history [36].

This specific disposal of vTNFRs among viral species seems to be the result of their co-evolution with their hosts and would indicate that poxviruses have adapted their anti-TNF strategies to the specific TNF response they need to counteract. For example, although CrmB orthologues encoded by CPXV, MPXV, and VARV show high sequence similarity, they exhibit very different ligand binding properties. CrmB from VARV is the most effective at binding and inhibiting human TNF [69]. Similarly, CrmD from ECTV binds mouse TNF with high affinity, while its affinity for human TNF is lower [36,37].

It is also remarkable that CrmC and CrmE can bind both human and mouse TNF with high affinity, but they are only able to inhibit the biological activity of mouse TNF and human TNF, respectively. This may be explained by specific differences in how vTNFRs recognize their ligands, as binding nonessential residues may increase affinity but may not interfere with the cellular recognition, thus being irrelevant for neutralizing activity [36,52]. Regarding LTα, both CrmB and CrmD are able to interact with human LTα (hLTα) and murine LTα (mLTα), but CrmB shows higher affinity for the human ligand while CrmD prefers the murine one, supporting again the hypothesis that VARV and ECTV have adapted the binding specificity of vTNFRs to their specific hosts [36]. Interestingly, although both CrmB and CrmD can inhibit mLTα activity, only CrmB was shown to protect cells from hLTα-induced cytotoxicity. The level of specificity displayed by vTNFRs is very rare among members of the TNFRSF [70].

#### 2.1.2. Modulation of tmTNF

vTNFRs are considered more similar to TNFR2, as they share more sequence similarity with the extracellular domain of TNFR2 (32%) than to the one of TNFR1 (22%). In addition, the structure of CrmE [41], the unique vTNFR structure available to date, further supports this hypothesis. The fact that tmTNF was the main ligand for TNFR2 led to the exploration of the ability of vTNFRs to bind and block tmTNF activity. As a result, CrmB, CrmC, CrmD and CrmE have also been described as modulators of tmTNF activity, preventing its interaction with the cellular receptor [71].

Most of the tmTNF activity overlaps with sTNF, however tmTNF can additionally trigger reverse signalling after interaction with TNFR at the cell surface and dampen inflammatory processes [1,16,38,40,72]. In the context of viral immune modulation, vTNFRs were not only demonstrated to interact with tmTNF and block its cytotoxic effect, but also to trigger reverse signalling reducing proinflammatory signalling pathways such as those regulated by NF-κB and JAK-STAT [39]. Therefore, vTNFRs can modulate inflammation by either limiting the availability of sTNF or decreasing inflammatory cytokine production through reverse signalling after binding to tmTNF (Figure 1). 

The particular, the behaviour of CrmE deserves a special mention, as it binds both murine sTNF and tmTNF with high affinity but can only inhibit murine tmTNF activity. These data suggest that the molecular structures from mouse tmTNF and sTNF are different and raise two possibilities to explain this surprising behaviour: either tmTNF and sTNF bind to CrmE through different residues, or these interacting residues are the same, but the biological activity of these cytokines relies on different sites. In any case, the contribution of tmTNF signalling to the defence against poxvirus infections in mice becomes evident due to the fact that the expression of recombinant CrmE surprisingly enhanced the virulence of a VACV strain lacking other vTNFRs [58]. 

#### 2.1.3. Addition of a Chemokine Binding Domain: SECRET

Besides their CRDs, CrmB and CrmD contain an additional C-terminal domain, termed Smallpox virus-Encoded Chemokine Receptor (SECRET), with the ability to bind with high affinity and block the activity of a particular set of chemokines involved in mucosal and skin inflammation. This domain shares no sequence similarity to cellular TNFRs or any other known cellular protein and represents an independent folding domain that can exert its function either independently or fused to a TNFR [33]. The modulation of immune cell recruitment by the SECRET domain plays an important role in the initial stages of ECTV infection [33,73]. However, it was demonstrated that the chemokine inhibitory activity of the SECRET domain is not a key virulence factor for mousepox on its own, as its role in pathogenesis only becomes evident when the virus simultaneously retains the TNF inhibitory ability. As later detailed, it has been proposed that a spatially and temporally coordinated blockade of TNF and chemokines by a single viral immunomodulator, as shown for CrmD, might serve as an excellent strategy to inhibit cell recruitment and modulate the immune response in vivo.

Although the SECRET domain is not related to any cellular gene, sequence analysis of poxviral genomes revealed three additional genes encoding separate SECRET domain-containing proteins (SCPs) [33]. Despite their low sequence similarity, these SCPs bind the same set of chemokines as the SECRET domain from CrmB, reinforcing the specific folding and modular nature of this domain. The structure of SECRET from CrmD [74] showed a beta-sandwich fold also present in other viral chemokine binding proteins and viral immunomodulators [75,76] to the extent that this folding has been named poxvirus immune evasion domain [77,78]. 

A fine characterization of the functional boundaries between CRDs and the SECRET domain from CrmD has recently confirmed that CRD4, although initially assigned to the TNF binding region, contributes to the SECRET domain-mediated chemokine binding. This incertitude was already hinted at when Asp167 and Glu169 residues in CRD4 from CrmD were identified as key chemokine-binding determinants in the structure of the SECRET domain–CX3CL1 complex [74]. In line with this, it was demonstrated that at least the second part of CRD4 in CrmD is not involved in TNF binding but is probably required for the high-affinity chemokine interactions of the SECRET domain [52].

#### 2.1.4. T2 Protein 

T2 protein was the first vTNFR identified in poxvirus [79] and to date is the unique vTNFR described in the Leporipoxvirus genus, which includes Myxoma virus (MYXV), a highly pathogenic poxvirus affecting European rabbits, and the closely related Shope fibroma virus. The T2 protein from MYXV (M-T2) is expressed early after infection to bind rabbit TNF with high affinity and block its biological activity, while T2 from Shope fibroma virus can additionally inhibit human TNF [42,80,81]. M-T2 shows significant sequence similarity to the extracellular region of TNFR2, containing four CRDs. While the first three CRDs are key for binding and inhibition of TNF, additional anti-apoptotic activity described in M-T2 relies exclusively on CRD1 and CRD2 [82,83]. Indeed, CRD1 from M-T2 also contains an analogue of the PLAD from human TNFRs, named viral PLAD (vPLAD), that can interact with its cellular analogues to form unresponsive heterotrimers. By this mechanism, the intracellular pool of M-T2 is able to inhibit infection induced apoptosis in T lymphocytes by sequestration of cellular TNFRI in a ligand-independent manner, thus contributing to virus replication [43,44,45]. Whether a vPLAD could exist in other vTNFRs is a question still to be elucidated, in part due to the limited structural data available. In this sense, the data from the solved structure of VACV CrmE was not conclusive since it revealed the existence of an incomplete vPLAD [41]. 

Given its dual activity as a TNF and apoptosis inhibitor, it is not surprising that M-T2 was addressed as an obvious virulence factor. Indeed, the infection of susceptible European rabbits with MYXV lacking both gene copies encoding M-T2 resulted in disease attenuation and diminished mortality, indicating a critical role for this vTNFR in the development of myxomatosis [42].

#### 2.1.5. vCD30

vCD30 was identified as a soluble viral version of the cellular receptor CD30, which is also known as TNFRSF8 and expressed by a small subset of activated T and B cells [84]. vCD30 is secreted after CPXV and ECTV infection as a 12kDa protein containing two CRDs [46,85], and an orthologue has been identified in the deerpox virus genome [86]. vCD30 binds CD30 ligands (CD30L, also known as CD153) with very high affinity, preventing the interaction of CD30L with its cellular receptor and therefore interfering with the stimulation of T and B cells [46,85]. Additionally, vCD30 was the first vTNFR described to induce reverse signalling, promoting IL-8 expression through cell surface bound CD30L expressed on neutrophils [46]. Furthermore, vCD30 has been shown to modulate the Th1 inflammatory response in a mouse model of granuloma induction by inhibiting IFNγ production in splenocytes (Figure 1) [46]. Unexpectedly, despite its potential role in viral immune modulation, vCD30 was not found to affect virulence, since its deletion from the ECTV genome did not appear to impact mousepox pathogenesis [87].

### 2.2. vTNFBPs

In addition to vTNFRs, some poxvirus species have developed their own vTNFBPs to impede TNF signalling. The 2L protein was the first vTNFBP, with no sequence homology to any known cellular TNFR, identified from Tanapox virus (TPXV)-infected cell supernatants. Later, two homologs were found in the other two existing yatapoxvirus: Yaba-like disease virus (YLDV) and Yaba monkey tumor virus (YMTV). Similar to some vTNFRs, 2L binds human TNF with very high affinity to prevent cytokine signalling through TNFR1 and TNFR2 [88], however, unlike vTNFRs, 2L shows sequence similarity limited to a region of the class I major histocompatibility complex (MHC-I) heavy chain [48]. Indeed, the crystal structure of the TPXV 2L complexed with TNF confirmed structural similarities with MHC-I molecules [49] and another study described its ability to complex with human β2 microglobulin [47]. Since this protein is required for cellular MHC I antiviral function, it has been suggested that TPXV may use this interaction to evade cellular immune responses. A similar strategy has been reported in cytomegalovirus and molluscum contagiosum virus, as they also encode MHC class I homologs that can complex with β2 microglobulin to impair cellular recognition of virus-infected cells [89,90,91]. 

This structural analysis also showed that the 2L interaction with TNF resembles the TNF interaction with the cellular TNFR, a central TNF trimmer bound by three symmetrically arranged 2L molecules. These data raise the hypothesis that 2L might have originated from a fragmented gene encoding an MHC-I molecule that was subsequently incorporated and modified by poxviruses to bind and block TNF.

Besides the 2L protein from yatapoxviruses, proteins SPV003 and DPV008 from swinepox virus and deerpox virus, respectively, are also considered as vTNFBPs [86,92]. In consonance with vTNFRs, the ligand specificity of vTNFBPs seems to be somehow related to the correspondent virus tropism. For example, TPXV-2L exhibits broader specificity and high affinity for human, monkey and canine TNF, while the swine-specific SPV003 only binds porcine TNF with high affinity [48]. These differences suggest that some specific residues from each TNFBP in their TNF binding domains dictate their precise specificity, indicating that, similarly to vTNFRs, vTNFBPs have evolved to meet the needs imposed by a particular host defence.

## 3. Relevance in Poxvirus Pathogenesis

Although we have previously mentioned the relative importance of CD30 and T2, the contribution of vTNFRs to poxvirus pathogenesis was initially unclear, as most reports generating viruses lacking expression of vTNFRs showed very limited effects or were not conclusive. For example, deletion of CrmB resulted in an attenuated phenotype compared to parental virus in an intracranial model of CPXV [34], a route of infection not natural for poxviruses. Another limitation of this study is that the attenuated phenotype cannot be attributed solely to the inactivation of CrmB since the selection of inadvertent mutations elsewhere in the viral genome was not controlled with the construction of a revertant virus or by sequencing the complete viral genome. The deletion of CrmE and CrmC in VACV USSR strain led to moderate attenuation of the viral phenotype in animal models [58]. The same study also tried a different approach, incorporating genes encoding CrmB, CrmC or CrmE from CPXV to a highly attenuated VACV (Western Reserve strain with deletion of the thymidine kinase), which caused a minimal increase in virulence. In general, the administration route and the high viral doses inoculated in these studies did not mimic the natural modes of virus transmission and may limit the conclusions obtained on disease pathogenesis. Moreover, the presence of additional active vTNFRs in the genomes of the recombinant viruses used could influence the results and mask the real contribution of a given vTNFR. 

In contrast, CrmD is the only functional TNF inhibitor in ECTV, a strict mouse pathogen causing mousepox disease, making ECTV infection of susceptible mice a suitable model to study the contribution of this vTNFR to poxvirus pathogenesis. In contrast with the observations described above, the infection of susceptible BALB/c mice with an ECTV lacking CrmD expression (ECTV∆CrmD), and therefore in the absence of any TNF blocking activity, resulted in a dramatic virus attenuation and a difference of six orders of magnitude in LD50 compared to wild type virus [73]. In the absence of CrmD ECTV infection induced a strong NK and CD8+ T cell-based response, which impaired viral replication in the liver and spleen, reducing mortality. These findings not only addressed CrmD as a key virulence factor for mousepox but also support a role for TNF in anti-viral defence, and raise the possibility that CrmB, the only vTNFR encoded by VARV, might have a similar potent contribution to smallpox pathogenesis. 

TNF modulation by ECTV represents an excellent example illustrating how virus evasion strategies can increase the understanding of the host immune regulation mechanisms. In this sense, the analysis of the specific contribution to mousepox pathogenesis of CRDs and SECRET domains from CrmD revealed that chemokines and TNF cooperate during virus infection to develop the inflammatory response and provide anti-viral immunity. Thus, the reinsertion of either the anti-TNF (CRDs domain) or the anti-chemokine (SECRET domain) activities into ECTV∆CrmD did not restore full virulence, indicating that both functions need to operate together to evade the potent antiviral response. 

In line with this, a recent study evidenced the deleterious consequences of an excessive TNF based response during poxvirus infections. The intranasal infection of mouse strains genetically resistant to mousepox with ECTV∆CrmD caused uniform mortality due to excessive TNF action and a dysregulated inflammatory response [39]. In this case, the blockade of TNF, IL-6 or IL-10R with monoclonal antibodies reduced the lung pathology and restored the resistance to infection (60 to 100% survival). In contrast, TNF blockade by CrmD during wild type infection resulted in a reduction in pathology, leukocyte recruitment, and inflammatory cytokine production in the lungs. Although the action of CrmD in favour of the host seems counterintuitive, it has been proposed that enabling survival would also facilitate viral spreading as an advantage to the virus. In conclusion, the role of CrmD in the mousepox model of infection confirms previous observations indicating a prominent impact of vTNFRs on poxvirus pathogenesis.

## 4. Therapeutic Use of vTNFRs

Dysregulated TNF production has also been postulated as being responsible for the pathology in many inflammatory disorders, such as rheumatoid arthritis, psoriasis, vasculitis or Crohn's disease [93]. In these cases, approved therapies for human use involve different TNF inhibitors, including four monoclonal anti-TNF antibodies and a soluble version of the human TNFR2 named etanercept [94,95]. However, therapeutic TNF blockade is still not accurate, and often these anti-TNF treatments lead to serious and diverse effects that can even result in life threatening conditions, such as an increased risk of infections or tuberculosis reactivation [96,97]. In this context, and similarly to other viral decoy receptors, the diverse strategies adopted by poxviruses to evade TNF can help us to develop new TNF inhibitors or to improve the existing ones by the incorporation of immunomodulatory features from poxvirus inhibitors [98].

Some vTNFRs show broader ligand specificity or are more potent inhibitors of TNF signalling than etanercept [36], supporting a therapeutic use for these viral proteins. For example, epicutaneous administration of recombinant VARV CrmB has been able to revert the TNF induced migration of skin leukocytes and colony forming activity of bone marrow cells in vivo, including an experimental mouse model of contact dermatitis [99,100]. Even the injection of DNA encoding CRDs from VARV CrmB appeared to slightly diminish the severity of the pathology in a rat model of collagen-induced arthritis [101].

**Figure 2:**
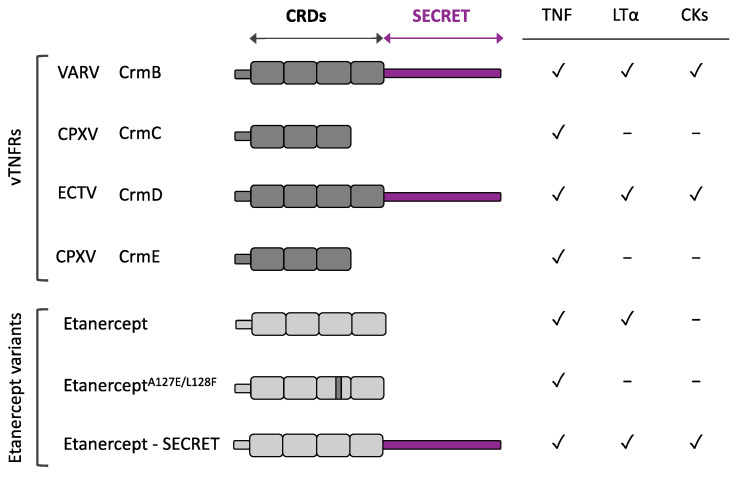
Etanercept variants inspired by vTNFRs. Schematic representation of the different domains and human ligand binding specificities from selected vTNFRs and etanercept, the human soluble TNFR clinically used to treat chronic inflammatory disorders. Anti-TNF and anti-chemokines activities rely on CRDs and SECRET domains, respectively. Modifications in etanercept, inspired by vTNFR CrmD, to generate a TNF inhibitor with impaired anti-hLT activity (EtanerceptA127E/L128F) or with anti-chemokine properties (Etanercept-SECRET) are depicted. CKs: chemokines.

Chemokines are also considered an important target for the development of anti-inflammatory therapies, as they mediate leukocyte migration [102]. As described above, CrmD combines both anti-TNF and anti-chemokines activities in a single molecule [73]. Inspired by this feature, a similar modification of etanercept, fusing it to a poxviral SECRET domain, has recently been accomplished (Figure 2). This chimeric protein TNFR2-SECRET showed high affinity for both TNF and chemokines, achieved protection from TNF effects, and was able to prevent chemokine-induced migration. Notably, this fusion protein was tested in a mouse model of arthritis with similar results to etanercept in delaying the development of clinical signs, even when using a smaller dose than the one of the established drug [103]. This report constitutes a proof of concept for new therapies involving etanercept-based bifunctional fusion constructs that develop a coordinated blockade of TNF and chemokines.

CrmD has also inspired an additional modification related to ligand specificity intended to prevent some of the adverse effects from actual anti-TNF therapies (Figure 2). Etanercept not only inhibits TNF but also human LTα and LTβ, which under some circumstances may account for some of the adverse effects described for etanercept [36,104,105]. On the contrary, CrmD is unable to block human LTα and an extensive analysis of CrmD ligand binding specificities revealed a structural difference with etanercept: the presence of a specific Glu-Phe-Glu motif in the 90s loop from CRD3. Transfer of this motif to etanercept was sufficient to reduce its anti-LTα activity more than 60-fold while only weakening its TNF blocking capacity three-fold [52]. Therefore, this modified protein could represent a safer alternative TNF inhibitor with fewer adverse effects than conventional etanercept.

vTNFRs could have an additional application in poxvirus vaccination. Although current vaccines against virulent poxviruses have proven their efficacy in the past, there is still uncertainty about the duration of their immunity and their adverse effects, raising considerable interest to develop new and safer vaccines. In this sense, immunization with CrmD protects mice from otherwise lethal mousepox, most likely due to antibody-mediated blockade of CrmD [73]. A similar result was obtained after immunization with another well-defined virulence factor, the ECTV type I IFN binding protein [106]. Interestingly, some well-established VACV vaccines, like Dryvax and Modified VACV Ankara, lack CrmB expression and encode a truncated version of the type I IFN binding protein [107,108,109]. Thus, it has been proposed that virus neutralization could additionally be achieved by targeting key immunomodulatory viral proteins to develop a new generation of vaccines.

In conclusion, these soluble decoy receptors constitute a highly efficient strategy to prevent TNF effects, as deduced from the recent evidence demonstrating the relevant contribution of vTNFRs to poxvirus virulence and pathogenesis. Thus, further research on this repertoire of TNF decoy receptors might inspire additional modifications to improve current therapies for inflammatory diseases, or even the generation of new anti-inflammatory molecules with a viral origin. 

## Figures and Tables

**Table 1:** Poxvirus encoded TNFRs and TNFBPs.

	Protein	Key Refs.	Virus	Virulence Factor	Known Ligands	Cellular Homology	kDa	Special Traits
vTNFRs	CrmB	[33,34]	CMPXV, CPXV, MPXV, VARV	Unclear (~50-fold LD_50_ reduction after intracranial inoculation)	**hTNF**, mTNF, **hLTα**, mLTα	TNFR2	48	SECRET domain-mediated chemokine inhibition; Reverse signalling
	CrmC	[35,36]	CPXV, VACV	Minor effect (difference in weight loss)	hTNF, **mTNF**	TNFR2	25	Reverse signalling
	CrmD	[37,38,39,40]	CPXV, ECTV	Yes (reduced LD_50_ in 6 orders of magnitude)	hTNF, **mTNF**, hLTα, **mLTα**	TNFR2	46	SECRET domain-mediated chemokine inhibition; Reverse signalling
	CrmE	[41]	CPXV, VACV	Minor effect (difference in weight loss)	**hTNF**, mTNF	TNFR2	18	Mouse tmTNF inhibition w/o sTNF; Reverse signalling
	T2	[42,43,44,45]	MYXV	Yes (reduce mortality)	**rabbit TNF**	TNFR2	M-T2: 40.5	PLAD mediated antiapoptotic activity
			SFV		**hTNF, rabbit TNF**	TNFR2	S-T2: 58	
	vCD30	[46]	DPXV, CPXV, ECTV	No	**CD30L**	CD30	12	Reverse signalling through CD30L; inhibition of IFNγ production in splenocytes
vTNFBPs	2L	[47,48,49]	TPXV	ND	**rabbit, human, monkey, canine TNF**	MHC I heavy chain	47	Can associate with β2 microglobulin to inhibit MHC-I
			YMTV		**rabbit, human, monkey TNF**			
	SPV003	[48]	SPXV	ND	**porcine TNF**	MHC I heavy chain	47	−

ND, not determined; CPXV, cowpox virus; CMPXV, camelpox virus; DPXV, deerpox virus; ECTV, ectromelia virus; MPXV, monkeypox virus; MYXV, myxoma virus; SFV, shope fibroma virus; SPXV, swinepox virus; TPXV, tanapoxvirus; VARV, variola virus; VACV, vaccinia virus; YMTV, yaba monkey tumour virus; hTNF, human TNF; mTNF, mouse TNF. Underlining indicates ligand blocking activity by the corresponding viral decoy receptor.
